# Incomplete tuberculosis reporting and registration to the surveillance system in southwestern China of Yunnan Province: an inventory survey

**DOI:** 10.1186/s12889-024-18794-2

**Published:** 2024-05-25

**Authors:** Jinou Chen, Yubing Qiu, Wei Wu, Ying Pan, Rui Yang, Ling Li, Yunbin Yang, Kunyun Lu, Lin Xu

**Affiliations:** 1 Yunnan Center for Disease Control and Prevention, Kunming, China; 2 Kunming Medical University, Kunming, China; 3 Division of tuberculosis control and prevention, Yunnan Center for Disease Control and Prevention, 158# Dongsi Road, Xishan District, Kunming, 650000 Yunnan Province China

**Keywords:** Tuberculosis, Surveillance, Report, Register

## Abstract

**Background:**

The real-world tuberculosis (TB) surveillance data was generally incomplete due to underreporting and underdiagnosis. The inventory study aimed to assess and quantify the incompletion of surveillance systems in southwestern China.

**Methods:**

The inventory study was conducted at randomly selected health facilities (HF) by multi-stage stratified cluster sampling. The participants were included in the period between August of 2020 in province-level and prefecture-level HF, and in the period between June to December of 2020 in other categories of HF respectively. The clinical committee confirmed medical records were matched to the National Notifiable Disease Reporting System (NNDRS) and the Tuberculosis Information Management System (TBIMS) to define the report and register status. The underreporting and under-register rates were evaluated based on the matched data, and factors associated with underreport and under-register were assessed by the 2-level logistic multilevel model (MLM).

**Results:**

We enrolled 7,749 confirmed TB cases in the analysis. The province representative overall underreport rate to NNDRS was 1.6% (95% confidence interval, 95% CI, 1.3 − 1.9), and the overall under-register rate to TBIMS was 9.6% (95% CI, 8.9–10.3). The various underreport and under-register rates were displayed in different stratifications of background TB disease burden, HF level, HF category, and data source of the medical record in HF among prefectures of the province. The intraclass correlation coefficient (ICC) was 0.57 for the underreporting null MLM, indicating the facility-level cluster effect contributes a great share of variation in total variance. The two-level logistic MLM showed the data source of medical records in HF, diagnostic category of TB, and type of TB were associated with underreporting by adjusting other factors (*p* < 0.05). The ICC for under-register was 0.42, and the HF level, HF category, data source of medical records in HF, diagnostic category of TB and type of TB were associated with under-register by adjusting other factors (*p* < 0.05).

**Conclusion:**

The inventory study depicted incomplete TB reporting and registering to NNDRS and TBIMS in southwestern China. It implied that surveillance quality improvement would help advance the TB prevention and control strategy.

## Introduction

Tuberculosis (TB) is an airborne chronic communicable disease caused by the agent of *mycobacterium tuberculosis*, often invading lung tissue resulting in pulmonary tuberculosis (PTB). The World Health Organization (WHO) estimated that 10.6 million people will develop TB in 2022 [1], an increase of 2.9% and 6.0% from 10.3 to 10.0 million people in 2021 and 2020, respectively [2, 3], worse still, this was the highest newly reported number of diagnosed TB since the WHO began global surveillance from 1995. It implies the number of underreported, undiagnosed and untreated TB has grown, which resulted in more active TB cases and more serious community transmission during the epidemic of coronavirus disease 2019 (COVID-19) [4].

The surveillance of the notifiable disease was the key to disease control and prevention, it could provide the number and incidence of TB disease. Sensitive and accurate surveillance was crucial for epidemiological monitoring, statistical analysis, health resource allocation and public health decisions. Though the effectiveness and quality of surveillance depend on the complete and precise data, the real-world surveillance data was generally incomplete and inaccurate due to underdiagnosis and underreporting [5]. According to the WHO report, China ranked sixth in the order of the size of the gap between notified TB cases and the estimated TB incidence in ten countries, it was partially attributed to the combination of underreporting of diagnosed cases and underdiagnosis [3].

The web-based TB surveillance system in China was developed and applied for two decades. The National Notifiable Disease Reporting System (NNDRS) was introduced by Chinese Center for Disease Control and Prevention (CCDC) in the year of 2004 [6]. The main role of the NNDRS was monitoring and estimating the epidemic and outbreak for 40 kinds of infectious diseases including PTB. The presumptive, clinically diagnosed and laboratory confirmed PTB (contain tuberculous pleurisy) must report to NNDRS based on regulation. Subsequently, the Tuberculosis Information Management System (TBIMS) was built in the year of 2005 in accordance with the Chinese National TB Program (CNTP) [7]. The main function of the TBIMS was registering and recording the information of referral and diagnosed TB cases’ demography, the diagnoses and the follow-up of treatment. These two systems were independent and complementary to each other, they exchanged information through the diagnosed TB patients. The coverage of NNDRS included the TB designed and no-designated health facilities across the country, though the TBIMS only authorized to the designated health facilities.

Globally, studies have reported on the underreporting and under-registering of the surveillance system in many counties, though there were larger range and diversity estimations of the surveillance completeness under different scenarios of disease prevalence and health system condition [8–11]. Few nationwide studies assessed the accuracy and quality of surveillance data in China [12–14]. The previous study presented that 10.7% of patients were not reported to NNDRS and 30.9% of patients were not registered to TBIMS in the year 2016. However, due to the limited survey sample size, the heterogeneity of TB incidence and disease burden, and the difference in public health resource allocation and accessibility of health care service, the aggregated national study was far from precise. Additionally, whether the surveillance quality gradually improved over time was unknown. Thus, regional and detailed evidence was needed to clarify the surveillance accuracy and quality, the representative survey should be developed to assess the localized TB underreporting and under-registering.

We conducted the present inventory study to quantify the underreporting to NNDRS and the under-register to TBIMS in southwest China. We also investigated the factors associated with TB patients underreporting and under-register to these two systems. The study aims to describe the gaps in the matched information across the routine surveillance platforms, to supply evidence of improving the surveillance quality, and to evaluate the key parameter of estimating the TB incidence and burden.

## Methods

### General setting

The inventory study was conducted in the fields of Yunnan province in southwestern China. Yunnan shares the borderline with Vietnam, Laos and Myanmar. There were 16 prefectures and 129 countries in the province. The TB prevalence of Yunnan was moderate, the reported and registered TB rate was 60.1 and 51.0 per 100,000 population in the year 2020, respectively [15].

The TB delivery system of Yunnan was the “three-in-one” integrated model (TIOIM). Under this integrated model, the Center of Disease Control and Prevention (CDC) was in charge of comprehensive management for TB control, the TB designated health facility (DHF) provided TB diagnosis and treatment service, community-level healthcare institutions were responsible for referral the presumptive patients and follow-up the treatment of TB patients, specifically, the multi-drug resistant tuberculosis (MDR-TB) patients were treated at prefecture-level or provincial-level designated facilities [16]. Additionally, the non-designated health facility (NDHF) was in charge of referral the presumptive patients with abnormal chest X-ray (CXR) to DHF for further diagnosis and treatment.

The ideal TB disease reporting and registering process under TIOIM was as follows: a reporting card of TB disease was used by the physicians in DHF or NDHF for collecting the patients’ information during their initial visit, then the checked information had to report to NNDRS by the first consultant physicians within 24 h from initial visiting. The NDHF patients completed the referral process and arrived at the DHF TB clinic, or the DHF patients of other departments reached the TB clinic at DHF, while these patients were clinically diagnosed or laboratory confirmed by DHF, their information should be registered to the TBIMS for case treatment and follow-up management.

### Study design

In brief, we conducted a retrospective inventory investigation to quantify the patients underreporting to NNDRS and under-register to TBIMS, we also identified the demographic and other factors associated with the underreporting and under-register.

We constructed the primary database by extracting the information on outpatient records, inpatient records, radiographs and imaging records, and laboratory records from different departments of the study sites, since then, the diagnostic review was carried out by a group of independent qualified clinical experts. The reviewed records were matched with the NNDRS and TBIMS databases to check the reporting and registering status.

### Study sites and sampling

The study was conducted in sites of health facilities (HF). We categorized the facilities into DHF and NDHF according to the responsibility under TIOIM. Meanwhile, we divided HF into three categories according to their levels: province-level, prefecture-level, and county-level facilities respectively. In general, there were eight types of HF in Yunnan. The research collected the primary data from the randomly selected HF. The investigated HF was distributed in 16 prefectures of the province: Baoshan, Chuxiong, Dali, Dehong, Diqing, Honghe, Kunming, Lijiang, Lincang, Nujiang, Puer, Qujing, Wenshan, Xishuangbanna, Yuxi, Zhaotong.

The research aimed to obtain representative samples of the whole province, thus, the multi-stage stratified cluster sampling approach was used. The sampling cluster was defined as the investigated health facilities. The first-stage sampling was randomly sampled out the counties as the study sites, the basis of this stage was the expected number of counties which were weighted by the TB disease burden and incidence for different regions. Then we conducted the second-stage sampling, we randomly chose one DHF and one NDHF from the province-level facilities. We defined prefecture-level facilities in the third-stage sampling, we randomly selected one DHF and one NDHF from each of the 16 prefectures of the province. The fourth stage was for sampling out the county-level facilities, we randomly sampled out the expected number of DHF and NDHF from each of the counties selected in the first stage of sampling, we determined the expected sample size based on the historical number of reported cards in facilities.

### Participants

We included the participants who met the eligibility criteria of the study. The eligible participants were those diagnosed with PTB, tuberculous pleurisy (TBP), and MDR-TB in study sites. The diagnosis was in accordance with the national diagnostic standard of CNTP. The eligible participants were included in the period between August of 2020 in province-level and prefecture-level DHF, and in the period between June to December of 2020 in other categories of health facilities respectively.

We excluded the presumptive TB patients, old tuberculosis, nontuberculous mycobacteria (NTM) disease, and other lung disease.

### Data collection and variables

We exported the medical records (MR) by using the HIS (hospital information system) and its sub-system, LIS (laboratory information system) and PACS (picture archiving and communication system) among the investigated health facilities. The time frame of the exported data was between July 1, 2020 and December 31, 2020. The MR of participants’ level data containing the information included: name, age, gender, national identification number, diagnostic category of TB (clinically diagnosed, bacteriological negative, bacteriological positive), and type of TB (PTB, TBP, MDR-TB). The criterion of extracting MR were these MRs mentioned “tuberculosis” or “TB”.

The investigated health facilities information containing: background burden of TB disease in the area of the investigated health facilities (low, TB prevalence lower than 40 per 100, 000 population; moderate, TB prevalence between 40 and 100 per 100, 000 population; high, TB prevalence over 100 per 100, 000 population), the level of health facilities (province-level, prefecture-level, county-level), health facilities categories (DHF, NDHF), data source of MR in health facilities (outpatient, laboratory, inpatient).

We extract the TB cases reporting data from NNDRS in a time interval between January 1, 2019 and June 30, 2021, the key variables containing the name, age, gender, national identification number, NNDRS identification number, date of illness onset, date of diagnosis and other patient characteristics. Meanwhile, we collected the TB registering data from the TBIMS in the same time interval. The following information was included: name, age, gender, national identification number, TBIMS identification number, date of diagnosis, date of treatment, and other patient features.

### Clinical review and key definition

After the data collection steps, we excluded the duplicated records and the residence records out of the province in the medical records dataset. We set up a diagnostic committee consisting of a group of clinical experts to review the diagnosis and CXR results of all the medical records. We excluded the old tuberculosis, NTM disease and other lung diseases according to the committee experts’ opinion.

We merged these three datasets of NNDRS reporting data, TBIMS registering data, and the experts reviewed MR data. The main index of the matching dataset was the name and national identification number of participants in experts reviewed MR data. Those participants who had identical names and national identification numbers in both MR and NNDRS, and appeared simultaneously in two datasets were the reported cases, otherwise only in MR were the underreported cases. Those participants who had identical names and national identification numbers in both MR and TBIMS, and appeared simultaneously in two data sets were the registered cases, otherwise only in MR were the under-register cases.

### Statistical analysis

All statistical analysis was conducted with R software version 4.0.2. The level of *P* < 0.05, two-sided was set as statistical significance.

Participants’ characteristics were grouped based on the report and register status; the classified variables were counted according to frequency. The rate and its 95% confidence interval (95% CI) were best estimated with the exact Beta Distribution [17]. The comparisons between groups were using the Chi-square test and Fisher’s exact test as appropriate.

#### The multilevel model

Multilevel model (MLM) is becoming the standard method of analyzing nested and hierarchical data, it is also known as hierarchical linear model (HLM) or linear mixed model (LMM) [18]. In our study, the different individual participants (level 1) were nested within multiple health facilities (level 2). Considering this “individual-facility” hierarchical structure data, there were nonnegligible intraclass correlation effects of participants within the same facility and between-class variation effects of facilities. Therefore, we built the following two-level logistic MLM to explore the influence factors associated with participants as level 1 and facilities as level 2:

$${Y}_{ij}=\text{log}(\frac{{\pi }_{ij}}{1-{\pi }_{ij}})={(\beta }_{0}+{\beta }_{0j}){+{{(\beta }_{1}+\beta }_{1j})X}_{ij}+{\mu }_{0j}+{\epsilon }_{ij}$$

$$\mu_{0j}\sim N(0,\tau_u^2\;),\;\varepsilon_{ij}\sim N(0,\sigma_e^2)$$

Where the *j* represents the facility level (level 2), *i* represents the participant’s level (level 1), ${Y}_{ij}$ represents the logit transformed report and register status, ${\beta }_{0}$is the fixed intercept of the model, ${\beta }_{0j}$ is the random intercepts at level 2; ${\beta }_{1}$ is the fixed coefficients of the model, ${\beta }_{1j}$ is the random slope at level 2; ${\mu }_{0j}$ is the effect of level 2 random residual, corresponding to the between-facilities variance ${\tau }_{u}^{2}$, ${\epsilon }_{ij}$ is the individual residual at level 1, corresponding to the within-facility variance ${\sigma }_{e}^{2}$.

We used the intraclass correlation coefficient (ICC, *ρ*) to evaluate the facility-cluster effects. It could be calculated by ${{\rho =\tau }_{u}^{2}/{(\tau }_{u}^{2}+\sigma }_{e}^{2})$. It is the percentage of total variance in outcome that may be attributed to facility-level variations. It may alternatively be conceptualized as the measurement of the correlation between two randomly chosen participants grouped within the same facility. Higher *ρ* indicated a greater share of outcome variation was associated with facility membership.

To establish the MLM for the study, we first built a null model that only involved the level 2 random intercept term. Then we check whether the level 2 facility effects were statistically significant. After that, we introduce explanatory variables into the model. We sequentially evaluated every single factor associated with the outcome of underreporting and under-register by univariate analysis. To build the multivariate model, we first analyzed the correlation between variables aiming to avoid the multicollinearity introduced to the multiple variables model. Then the unrelated variables were included in the multivariable analysis, we evaluated the goodness of model fit by the Akaike’s information criterion (AIC) value.

## Results

### The inventory study and the characteristics of participants

**Fig. 1:**
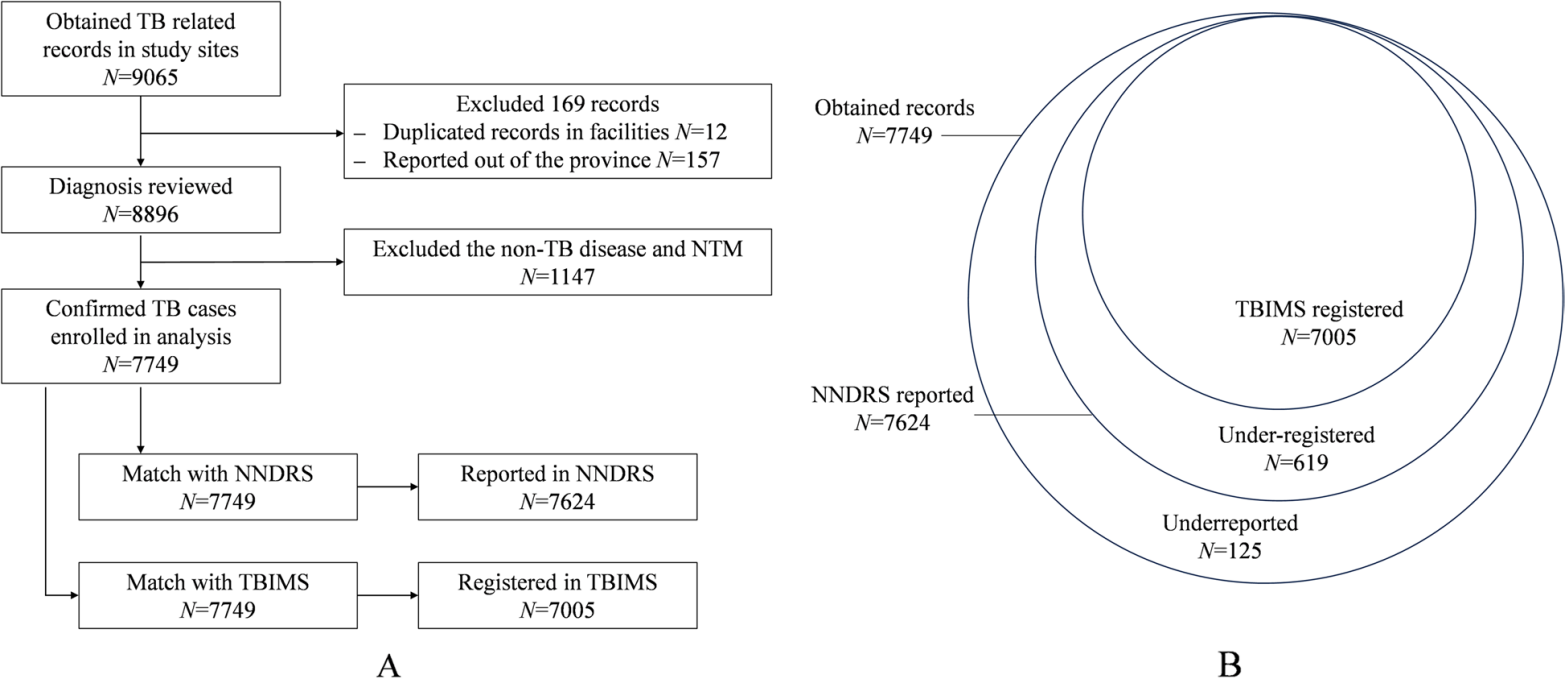
The flowchart and Venn diagram of the study. **A** The flowchart of the study. **B** The Venn diagram of the relationship for the obtained medical records matched with NNDRS and TBIMS. Abbreviations: TB, tuberculosis; NTM, nontuberculous mycobacteria, NNDRS, National Notifiable Disease Reporting System; TBIMS, Tuberculosis Information Management System

At the individual level, we defined 9,065 MR in health facilities, we first excluded 169 records from the original data (12 duplicated cases between investigated facilities, and 157 records reported out of the province). Afterward, the diagnostic committee of experts conducted a diagnosis review, they examined the CXR and diagnosis of 8,896 MR, and they excluded 1,147 old tuberculosis, NTM and other lung diseases. Finally, we enrolled 7,749 confirmed TB cases in the analysis (Fig. 1A).

Of all the identified TB patients, 4,881 (63.0%) were males and 2,868 (37.0%) were females. There were 6,265 (80.8%) cases were 15–64 years of age, and 1,371 (17,7%) aged over 65 years, only 113 (1.46%) were under 15 years old. Most of the patients were bacteriological negative TB (3,068, 39.6%), and bacteriological positive TB (4,252, 54.9%), only 429 (5.5%) were clinically diagnosed cases. There were 7,047 (90.9%) active PTB cases, 428 (5.5%) were TBP; and 274 (3.6%) were MDR-TB.

**Fig. 2:**
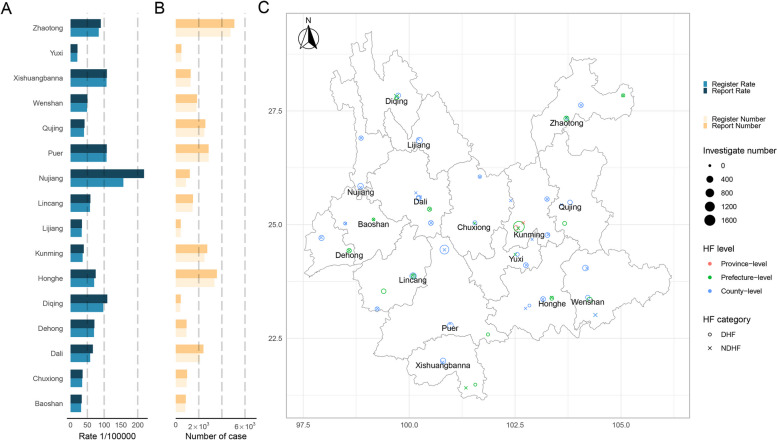
The brief information of investigation and the TB disease burden of study sites in Yunnan, China. **A** the reported and registered rate of TB in prefectures of Yunnan in 2020. **B** the reported and registered number of TB in prefectures of Yunnan in 2020. **C** the locations, levels, categories of investigated health facilities, and the enrolled number of participants. Abbreviations: TB, tuberculosis; HF, health facility; DHF, designated health facility; NDHF, non-designated health facility

In 16 prefectures of the province, the number of reported TB cases and report rate, the number of registered TB cases and register rate were demonstrated in Fig. 2A and B respectively. There were significant regional differences and heterogeneity of disease burden in the prefectures.

At the facility level, there were 73 health facilities participated in the study. These facilities had reported or registered TB cases during the time frame of the study. Overall, the number of medical records among these investigated facilities ranged from 50 to 1,979 cases (Fig. 2C). Of all the TB patients, there were 2,883 (37.2%) cases, 3,597 (46.4%) cases, and 1,269 (16.4%) cases from low, moderate, and high TB burden regions, respectively. There were 124 (1.6%) cases from province-level facilities, 2,975 (38.4%) cases from prefecture-level facilities, and 4,650 (60.0%) cases from county-level facilities. Most TB patients were from DHF (7,445, 96.1%), and 304 (3.9%) cases from NDHF. In the health facilities, the majority of cases were extracted from outpatient records (5,472, 70.6%), the rest of the 808 (10.4%) cases were extracted from the laboratory records, and 1,469 (19.0%) were extracted from the inpatient records.

### The overall underreporting and under-register

We matched the MR with NNDRS and TBIMS data, the relation of these datasets is shown in Fig. 1B. The overall underreport rate was 1.6% (95% CI, 1.3 − 1.9), and the overall under-register rate was 9.6% (95% CI, 8.9–10.3).

**Fig. 3:**
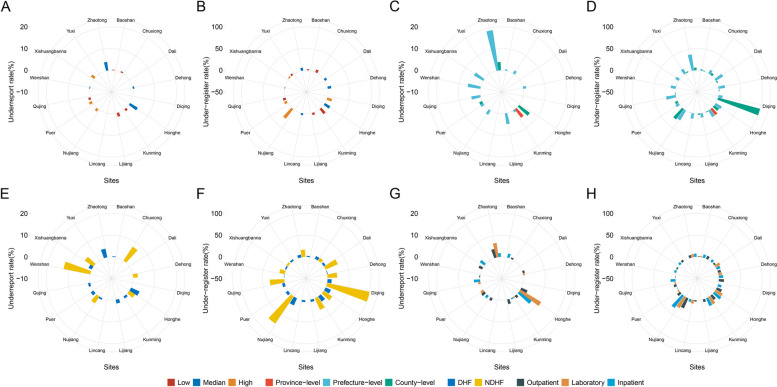
The circular plot of underreport and under-register TB at the facility level among prefectures in Yunnan, China. **A** the underreport rate stratified by prefectures and burden of TB disease. **B** the under-register rate stratified by prefectures and burden of TB disease. **C** the underreport rate stratified by prefectures and level of health facilities. **D** the under-register rate stratified by prefectures and level of health facilities. **E** the underreport rate stratified by prefectures and category of health facilities. **F** the under-register rate stratified by prefectures and category of health facilities. **G** the underreport rate stratified by prefectures and data source of MR in health facilities. **H** the under-register rate stratified by prefectures and data source of MR in health facilities. Abbreviations: TB, tuberculosis; HF, health facility; DHF, designated health facility; NDHF, non-designated health facility; MR, medical records

Prefectural heterogeneity of underreport and under-register of TB were observed (Table 1). Across 16 prefectures of the province, the range of underreport rate was 0% (95% CI, 0–0.9) to 4.2% (95% CI, 2.9–5.9), the under-register rate ranged from 1.1% (95% CI, 0.2–3.1) to 27.9% (95% CI, 22.9–33.3). The underreport and under-register rates varied greatly in different stratifications of TB disease burden, HF level, HF category, and data source of MR in HF among prefectures (Fig. 3).

**Table 1:** The characteristic of TB cases enrolled in the inventory study of Yunnan, China

Characteristic	Report	Underreport		Register	Under-register	
	*N*(%)		*P*	*N*(%)		*p*
Overall	7,624(98.4%)	125(1.6%)		7,005(90.4%)	744(9.6%)	
Prefecture						
Baoshan	328 (99.7%)	1 (0.3%)	< 0.01	321 (97.6%)	8 (2.4%)	< 0.01
Chuxiong	172 (99.4%)	1 (0.6%)		161 (93.1%)	12 (6.9%)	
Dali	720 (100.0%)	0 (0.0%)		681 (94.6%)	39 (5.4%)	
Dehong	283 (99.3%)	2 (0.7%)		262 (91.9%)	23 (8.1%)	
Diqing	96 (100.0%)	0 (0.0%)		85 (88.5%)	11 (11.5%)	
Honghe	713 (95.8%)	31 (4.2%)		638 (85.8%)	106 (14.2%)	
Kunming	1,958 (98.9%)	21 (1.1%)		1,688 (85.3%)	291 (14.7%)	
Lijiang	55 (98.2%)	1 (1.8%)		53 (94.6%)	3 (5.4%)	
Lincang	419 (100.0%)	0 (0.0%)		401 (95.7%)	18 (4.3%)	
Nujiang	301 (98.7%)	4 (1.3%)		220 (72.1%)	85 (27.9%)	
Puer	593 (98.5%)	9 (1.5%)		562 (93.4%)	40 (6.6%)	
Qujing	293 (99.0%)	3 (1.0%)		277 (93.6%)	19 (6.4%)	
Wenshan	283 (99.6%)	1 (0.4%)		281 (98.9%)	3 (1.1%)	
Xishuangbanna	261 (98.1%)	5 (1.9%)		257 (96.6%)	9 (3.4%)	
Yuxi	50 (100.0%)	0 (0.0%)		48 (96.0%)	2 (4.0%)	
Zhaotong	1,099 (96.0%)	46 (4.0%)		1,070 (93.4%)	75 (6.6%)	
Burden of TB disease			< 0.01			< 0.01
Low	2,856 (99.1%)	27 (0.9%)		2,548 (88.4%)	335 (11.6%)	
Median	3,517 (97.8%)	80 (2.2%)		3,333 (92.7%)	264 (7.3%)	
High	1,251 (98.6%)	18 (1.4%)		1,124 (88.6%)	145 (11.4%)	
HF level						
Province-level	118 (95.2%)	6 (4.8%)	0.01*	102 (82.3%)	22 (17.7%)	< 0.01
Prefecture-level	2,937 (98.7%)	38 (1.3%)		2,500 (84.0%)	475 (16.0%)	
County-level	4,569 (98.3%)	81 (1.7%)		4,403 (94.7%)	247 (5.3%)	
HF category						
DHF	7,329 (98.4%)	116 (1.6%)	0.06*	6,794 (91.3%)	651 (8.7%)	< 0.01
NDHF	295 (97.0%)	9 (3.0%)		211 (69.4%)	93 (30.6%)	
Data source						
Outpatient	5,384 (98.4%)	88 (1.6%)	0.60	5,009 (91.5%)	463 (8.5%)	< 0.01
Laboratory	792 (98.0%)	16 (2.0%)		728 (90.1%)	80 (9.9%)	
Inpatient	1,448 (98.6%)	21 (1.4%)		1,268 (86.3%)	201 (13.7%)	
Gender						
Male	4,800 (98.3%)	81 (1.7%)	0.67	4,411 (90.4%)	470 (9.6%)	0.91
Female	2,824 (98.5%)	44 (1.5%)		2,594 (90.4%)	274 (9.6%)	
Age(years)						
<15	108 (95.6%)	5 (4.4%)	0.01*	102 (90.3%)	11 (9.7%)	0.33
15–64	6,159 (98.3%)	106 (1.7%)		5,649 (90.2%)	616 (9.8%)	
>65	1,357 (99.0%)	14 (1.0%)		1,254 (91.5%)	117 (8.5%)	
Diagnostic category						
Clinically diagnosed	349 (81.4%)	80 (18.6%)	< 0.01	183 (42.7%)	246 (57.3%)	< 0.01
Bacteriological negative	3,055 (99.6%)	13 (0.4%)		2,866 (93.4%)	202 (6.6%)	
Bacteriological positive	4,220 (99.2%)	32 (0.8%)		3,956 (93.0%)	296 (7.0%)	
Type of TB						
PTB	6,931 (98.4%)	116 (1.6%)	0.21	6,356 (90.2%)	691 (9.8%)	0.01
TBP	420 (98.1%)	8 (1.9%)		387 (90.4%)	41 (9.6%)	
MDR-TB	273 (99.6%)	1 (0.4%)		262 (95.6%)	12 (4.4%)	

*Tested by Fisher’s exact test

*Abbreviations*: *TB *Tuberculosis, *HF *Health facility, *DHF* Designated health facility, *NDHF* No-designated health facility, *PTB* Pulmonary tuberculosis, *TBP* Tuberculous pleurisy, *MDR-TB* Multi-drug resistant tuberculosis

### Univariate analysis of factors associated with underreporting and under-register

**Fig. 4:**
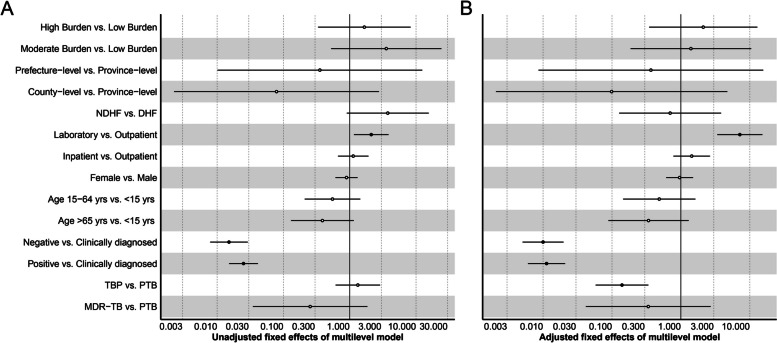
The fixed effects of factors associated with underreport TB in Yunnan, China. **A** the unadjusted fixed effects of factors associated with underreport TB. **B** the adjusted fixed effects of factors associated with underreport TB. Abbreviations: TB, tuberculosis; HF, health facility; DHF, designated health facility; NDHF, non-designated health facility; PTB, pulmonary tuberculosis; TBP, tuberculous pleurisy; MDR-TB, multi-drug resistant tuberculosis

The chi-square test showed a significant difference in underreported rate between different burdens of TB, HF level, and HF category, age of patients, and diagnostic category of TB (*p* < 0.05, Table 1). The ICC was 0.57 for the underreporting null MLM (Table 2), the nested data demonstrated the total underreporting variance was highly attributed to the diversity between different HF members. For the univariate MLM analysis, the associations of underreporting were statistically significant for the data source of MR in HF, and diagnostic category of TB (*p* < 0.05, Fig. 4A).

**Table 2:** The characteristic of null and full model of the multi-level logistic regression

	Fixed effect of intercepts				Random effect			
	Estimate	Std.error	*z*	*p*	${\sigma }_{e}^{2}$	${\tau }_{u}^{2}$	ICC	AIC
Underreport								
Null model^†^	-5.55	0.49	-11.28	< 0.01	4.29	3.29	0.57	1145.2
Full model^‡^	-0.45	1.88	-0.24	0.81				826.4
Under-register								
Null model^†^	-2.73	0.21	-12.74	< 0.01	2.41	3.29	0.42	4378.5
Full model^‡^	1.24	0.89	1.40	0.16				3705.0

^†^Null model contained the level-2 random intercept

^‡^Full model was adjusted for the burden of TB disease, level of health facilities, health facilities categories and data source of medical records in health facilities; and the age, gender, diagnostic category of TB, type of TB for the patients

*Abbreviations: ICC* intraclass correlation coefficient, *AIC* Akaike information criterion

The test showed that there was a statistically significant difference in under-register in the burden of TB, HF level, and HF category, data source of MR in HF, diagnostic category of TB and type of TB (*p* < 0.05, Table 1). The ICC for under-register was 0.42, indicating the variation of under-register was also mainly attributed to the difference between health facilities. The univariate MLM showed factors of the HF level, and HF category, data source of MR in HF, diagnostic category of TB and type of TB were associated with the under-register (*p* < 0.05, Fig. 4A).

### Multilevel model for factors associated with underreporting and under-register

In the pairwise correlation of dependent variables was not significant (correlation coefficients < 0.2, *p* > 0.05), the multicollinearity does not exist. The fixed scale null model showed the differences at level 2 were statistically significant (*p* < 0.05, Table 2), and the different random intercepts and strong intraclass correlation (ICC > 0.4) indicated that the two-level logistic MLM was appropriate for the study to depict the associations between factors and underreport and under-register in Yunnan.

The two-level logistic MLM showed that the data source of MR in HF, diagnostic category of TB, and type of TB were associated with underreporting (*p* < 0.05, Fig. 4B). The risk of underreporting for the MR source in the laboratory was about 7.2 times than the outpatient, the odds of underreporting were lower for bacteriological negative (adjusted odds ratio, aOR = 0.01, 95% CI, 0.01–0.02), bacteriological positive (aOR = 0.01, 95% CI, 0.01–0.02) compared with clinical diagnosed case, the TBP had a less likelihood of underreporting than PTB (aOR = 0.14, 95% CI, 0.06–0.34).

**Fig. 5:**
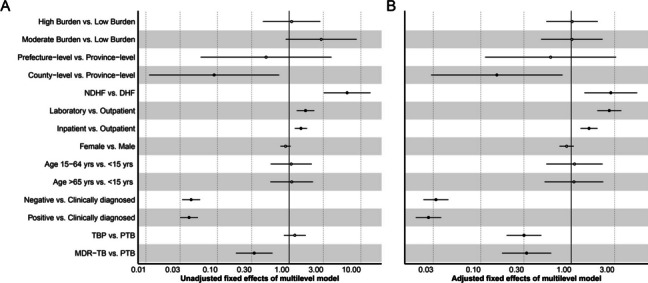
The fixed effects of factors associated with under-register TB in Yunnan, China. **A** the unadjusted fixed effects of factors associated with under-register TB. **B** the adjusted fixed effects of factors associated with under-register TB. Abbreviations: TB, tuberculosis; HF, health facility; DHF, designated health facility; NDHF, non-designated health facility; PTB, pulmonary tuberculosis; TBP, tuberculous pleurisy; MDR-TB, multi-drug resistant tuberculosis

The logistic MLM showed that the HF level, HF category, data source of MR in HF, diagnostic category of TB and type of TB were associated with under-register (*p* < 0.05, Fig. 5B). The patient diagnosis in NDHF (aOR = 2.73, 95% CI, 1.40–5.34), the MR source of laboratory (aOR = 2.63, 95% CI, 1.94–3.56) and inpatient (aOR = 1.57, 95% CI, 1.27–1,95) had a higher risk of under-register compared with the patient in DHF and the outpatient respectively. Meanwhile, the odds of under-register were lower for the country-level HF patients compared with the provincial HF (aOR = 0.15, 95% CI, 0.03–0.80). The bacteriological negative (aOR = 0.03, 95% CI, 0.02–0.04) and bacteriological positive (aOR = 0.03, 95% CI, 0.02–0.04) had lower under-register risk compared with the clinically diagnosed cases. The TBP (aOR = 0.30, 95% CI, 0.19–0.47) and MDR-TB (aOR = 0.32, 95% CI, 0.17–0.60) had a lower likelihood of under-register than PTB.

The different outcomes of TB underreporting and under-registering shared common influence factors of the data source of MR in HF, diagnostic category of TB and type of TB. The reduced AIC value of full logistic MLM for the underreporting and under-register compared with the null model indicated the multivariable model was the optimal solution (Table 2).

## Discussion

Our research examined the inventory approach based on the linkage between medical records of health facilities and the TB surveillance system of China. We described and quantified the underreporting and under-register of TB patients in health facilities under the TIOIM delivery system by using the matched cross-systems multiple-level data, meanwhile, we detailed the evidence for the quality of TB surveillance data in southwestern China. Additionally, we performed the appropriate assessment and documented the influence factors associated with the TB underreporting and under-register to the surveillance systems.

In the study, the result displayed that 1.6% of TB patients were underreporting to the NNDRS and 9.6% were under-register to the TBIMS. This uneven underreporting rate and under-register rate were mainly driven by the different roles of NNDRS and TBIMS in tuberculosis control. Under the TIOIM health care delivery system that includes the DHF and NDHF, the PTB patients were forced mandatory report in NNDRS by law on the prevention and control of infectious disease. However, only the patients in NDHF completed referral and made a definite diagnosis in DHF, or the patients directly sought healthcare in DHF were registered to the TBMIS. These two systems collected different aspects of patients’ information, namely the NNDRS obtained the critical demographic and diagnostic information for rapid notification and classification of the different infectious diseases, though the TBIMS caught the medical record information of examination and tests, the treatment and follow-up information specifically for TB disease. The gap between these two systems reflected the effectiveness of the referral and trace. For instance, for the patients who were identified by NDHF but not traced by DHF, the information might report to NNDRS but not register to TBIMS, whatever the diagnosis made by the NDHF. The difference showed there were 8% of patients still missing referral and trace information, they were diagnosed but were not included in the TB treatment and follow-up.

There was one-tenth of patients were missing for registering to the TBIMS, the under-register rate indicated that there might be a risk of disease control. The under-registered patients could not access health care service and reach the standard treatment regimen regulated by CNTP; the infectious source of under-registered active TB patients might contribute to persistent community transmission.

Globally, previous studies presented diverse underreporting and quality of surveillance systems under various study methodologies and settings. For the developed countries of Italy, Germany and South Africa with low TB burden, the underreporting rate ranged from 10 to 27%, though the rate would be higher for drug-resistant TB (DR-TB) patients at facilities [8–10]. Meanwhile, the developing countries with moderate and high disease burdens like India, Haiti, Puerto Rico and Kenya, the rate of not reported to routine surveillance systems was around 11–20% [11, 19–21]. In addition, the nationwide inventory study focused on the NTM disease in Saudi Arabia revealed a wide spectrum and geographic distribution of NTM, high prevalence of NTM diseases was uncovered by thorough investigation [22]. Further, the children’s TB was difficult to diagnose. Research in South Africa and Pakistan showed the reporting gaps were between 38% and 78% to surveillance systems [23, 24], which aggravated the vulnerability of the pediatric subgroup to TB disease. To summarize, the inventory studies presented a relatively high underreport rate worldwide regardless of study designs, greater disparities were presented for specific sub-populations. In the previous localized studies in China, Li et al. stated a series of inventory studies at the national level of the country. These studies concluded distinct underreporting and under-register rates, though the serial studies were entirely conducted in the years 2015 and 2016. The first reported study was conducted in 9 counties of 9 provinces in 2015, the study linked the health facilities records and social insurance system, which described a 19.3% under-register rate to TBIMS [13]. The second study included 984 health facilities in 32 provinces in the year 2015, the study narrated an 8.2% average under-register rate to TBIMS [12]. The third study was conducted in 6 counties among 6 provinces, the underreporting rate to NNDRS was 10.7% and the under-register rate to TBIMS was 30.9% in 2016 [14]. These large sample size studies reached obvious differentiation and unstable outcomes of the underreport and under-register rate. One possible speculation was not only the alternative designs and sampling methods, but the uneven background underreport and under-register among study fields. Although the multi-stage cluster sampling was used, the first two national studies only covered 9 and 6 provinces, the aggregated study might be biased by sampling coverage in different scenarios. Compared with our study, the underreport and under-register rate of Yunnan was 7 times and 2–3 times lower than the national average level respectively. There were three possible reasons for this relatively low rate. First, the TB information system and notification process were improved and systemic reshaped in years 2019 to 2020 [25, 26], and the underreport and under-register might be changed during years. Second, the background underreport and under-register of Yunnan might be lower than the mean level of other provinces in China, in other words, the quality of report and register to surveillance were superior in Yunnan. Third, by considering the TIOIM was extended during 2016 to 2017 in the province, the accessibility of TB related healthcare service was promoted greatly, the routine reporting and registering work might strengthen the coordination between NDHF and DHF and lessen the underreport and under-register.

Our study analyzed the factors associated with underreporting to NNDRS and under-register to TBIMS. The underreport was associated with the source of MR in HF, diagnostic category of TB, and type of TB, though there were more factors associated with under-register, including the HF level, HF category, data source of MR in HF, diagnostic category of TB and type of TB. The shared influence factors of underreport and under-register were the source of MR in HF, diagnostic category of TB, and type of TB. This suggested that the inpatients and laboratory-diagnosed patients were more likely not to report and register to these two systems, the counterintuitive result might be caused by the incomplete within-hospital referral process and the insufficient cooperation between different departments of DHF. It was noteworthy that the clinically diagnosed and PTB patients had higher odds of underreport and under-register, this indicated that the medical staff in HF should scrutinize these routine kinds of patients and their information must be collected and summarized to input to NNDRS and TBIMS, though medical staff would usually pay more attention to patients of whom the bacteriological diagnosis and specifical disease kinds of TBP and MDR-TB.

There were two added independent factors associated with under-register compared with factors associated with underreporting, which were HF level and HF category. The results showed the county-level HF had lower odds of under-register than the provincial HF, and the NDHF had higher odds than the DHF. Generally, under the TIOIM delivery system, the patients’ pathway of medical seeking was the tiered diagnosis and treatment model (TDTM), the core concept of TDTM was that the primary healthcare institution in charge of diagnosis and treatment of common diseases, while the main function of high-level facilities in charge of patients with acute and critical illness and patients with difficult and complicated diseases. In the context of our research, it implied that under this TIOIM and TDTM model, the shorter the patient’s pathway was, the less likely the patients were to be under-registered. The ideal full report and register model would be the decentralized diagnosis and treatment system, the patients would be notified and registered immediately while they are diagnosed and treated by decentralized capable primary healthcare institutions.

Our study analyzed the factors associated with underreporting and under-registering. The explanation of underreport and under-register might be associated with the undetected patients who are unable to access health services or were not diagnosed while seeking medical service. First, before the large-scale implementation of the molecular WHO-recommended rapid diagnostics (mWRDs) technology [27–29], the traditional diagnostic tools and diagnostic processes were insufficient to find patients promptly and accurately. The delay in diagnosis and treatment might be related to the low quality of surveillance information and prolonged patient pathways [30]. Second, the heterogeneity distribution of sociodemographics, culture, knowledge and stigma to patients among populations and regions [31, 32]. The most typical example was a special kind of minority nationality of the province serious discrimination against tuberculosis patients, leading to a lower willingness to seek medical treatment for these patients. Third, the shortage of trained medicine personnel, especially in the community healthcare center and primary health institutions, bring about the insufficient diagnostic capacity of the facility and the distrust between patients and healthcare provider. Fourth, the insufficient cooperation between NDHF and DHF, the patients were not traced and reached the DHF and they were undiagnosed. Moreover, the impact of COVID-19 on the health system led to the overloading of healthcare resources, the burnout while the waves of the epidemic resulted in the facilities not focusing on TB diseases.

The highlight and strength of the research included, firstly, the representative and well-designed study allowed us to depict the profile of TB reporting and registering to information systems for the whole province. Secondly, we enhanced the results by using the applicable analysis method to this matched hierarchical structured data. The model-free ICC coefficients were significant, meanwhile, the AIC for the logistic MLM model displayed superior performance, and they all demonstrated the optimal fitting to the special multilevel data. Third, the study analyzed 2-level MLM for facilities as the high level, the recent 2-level model was well-fitted, and the outcome was robust and explainable. Though the actual level-2 HF was nested within level-3 prefectures, the try-to-fit 3-level MLM did not converge because the overdispersion variance and variance-covariance matrix computed from finite-difference Hessian is not positive definite, and the small sample size in stratification makes it complex to interpret. Moreover, the study supported evidence-based public health decision-making. We could estimate the approximate TB incidence based on the underreporting rate, under-register rate, and notified TB rate, which reflected the solid TB burden in a specific region. Accordingly, this helped make regionally differentiated prevention and control strategies, the health resource relocation, supervision and surveillance quality improvement.

There were some limitations to the study. Firstly, the study was based on the patients who reached the facilities, the study design left out of consideration for the patients who did not seek healthcare service, even though this might contribute a share of underreporting and under-register. Future complementary inventory studies could validate the results by methods like the capture-recapture model, health-insurance matched data and linkage to drug consumption data [5, 8, 33]. Secondly, we did not include extra-pulmonary TB (EPTB), though the EPTB was not a notifiable infectious disease in China, the estimation of TB burden would be biased based directly on the underreporting and under-register rate. Other limitations like the study did not evaluate the COVID-19 pandemic impact on the completion of the surveillance. The NNDRS and TBIMS were updated in 2021 and 2022, the renewed system made a vision of a sustainable completeness surveillance network [34]. Hence, further research could reassess the dynamic surveillance quality.

## Conclusion

The representative inventory study documented the incomplete reporting and registering of TB to the infectious disease surveillance systems. We not only analyzed the gaps between systems but also offered proof of unbiased estimation for TB disease burden to support public health decision-making. The imperative system continuous upgrade, surveillance quality improvement and supervision should be conducted to bridge the information gap.

## Data Availability

No datasets were generated or analysed during the current study.

## Abbreviations

TB: Tuberculosis
HF: Health facilities
NNDRS: National Notifiable Disease Reporting System
TBIMS: Tuberculosis Information Management System
MLM: Multilevel model
ICC: Intraclass correlation coefficient
PTB: Pulmonary tuberculosis
WHO: World Health Organization
COVID-19: Coronavirus disease 2019
CCDC: Chinese Center for Disease Control and Prevention
CNTP: Chinese National TB Program
TIOIM: Three-in-one integrated model
CDC: Center of Disease Control and Prevention
DHF: Designated health facility
NDHF: Non-designated health facility
MDR-TB: Multi-drug resistant tuberculosis
CXR: Chest X-ray
HF: Health facilities
NTM: Nontuberculous mycobacteria
MR: Medical records
HIS: Hospital information system
LIS: Laboratory information system
PACS: Picture archiving and communication system
HLM: Hierarchical linear model
LMM: Linear mixed model
AIC: Akaike’s information criterion
DR-TB: Drug-resistant TB
TDTM: Tiered diagnosis and treatment model
mWRDs: Molecular WHO-recommended rapid diagnostics
EPTB: Extra-pulmonary TB
